# An electrically reconfigurable logic gate intrinsically enabled by spin-orbit materials

**DOI:** 10.1038/s41598-017-14783-1

**Published:** 2017-11-10

**Authors:** Mohammad Kazemi

**Affiliations:** Electrical and Computer Engineering Department, University of Rochester, Rochester, NY USA

## Abstract

The spin degree of freedom in magnetic devices has been discussed widely for computing, since it could significantly reduce energy dissipation, might enable beyond Von Neumann computing, and could have applications in quantum computing. For spin-based computing to become widespread, however, energy efficient logic gates comprising as few devices as possible are required. Considerable recent progress has been reported in this area. However, proposals for spin-based logic either require ancillary charge-based devices and circuits in each individual gate or adopt principals underlying charge-based computing by employing ancillary spin-based devices, which largely negates possible advantages. Here, we show that spin-orbit materials possess an intrinsic basis for the execution of logic operations. We present a spin-orbit logic gate that performs a universal logic operation utilizing the minimum possible number of devices, that is, the essential devices required for representing the logic operands. Also, whereas the previous proposals for spin-based logic require extra devices in each individual gate to provide reconfigurability, the proposed gate is ‘electrically’ reconfigurable at run-time simply by setting the amplitude of the clock pulse applied to the gate. We demonstrate, analytically and numerically with experimentally benchmarked models, that the gate performs logic operations and simultaneously stores the result, realizing the ‘stateful’ spin-based logic scalable to ultralow energy dissipation.

## Introduction

Spin degree of freedom has emerged as a primary candidate for the implementation of computing technologies that are nonvolatile and scalable to ultralow energy dissipation^1–3^. Materials with strong spin-orbit coupling, referred to as the spin-orbit materials, have been widely considered for efficient producing of spin current^4–14^. In-plane current injection into a spin-orbit layer gives rise to spin currents which produce torques, namely a damping-like spin-orbit torque and a field-like spin-orbit torque, on the magnetization of an adjacent ferromagnetic layer. Spin-orbit heterostructures have received significant attention, since for every electron charge injected into the spin-orbit layer, many $\tfrac{\hslash }{2}$ units of angular momentum may flow into the ferromagnet and produce spin-orbit torques on the magnetization, thereby providing an energy efficient mechanism for magnetization manipulation. Spin-orbit heterostructures with perpendicular magnetic anisotropy are the mainstay of spin-orbitronics owing to high thermal stability and scalability. Here, we show how current induced spin-orbit torques may inherently execute stateful logic operations in perpendicular-anisotropy heterostructures.

As in previous proposals for spin-based logic^15–19^, we represent data utilizing a bistable magnetization state. However, in contrast to proposal in ref.^15^ which utilizes current induced magnetic fields or the proposal in ref.^16^ which requires additional circuits to convert spin signals into magnetic fields for switching a nanomagnet, our work utilizes spin currents to directly switch nanomagnets via spin-orbit torques. Our proposal relies on an intrinsic property in spin-obit heterostructures to make possible a logic gate in which the same magnetic contacts that retain the logic inputs serve to simultaneously perform a logic operation and retain the result. This is in contrast to the structures proposed in refs^18,19^ which require ancillary magnetic contacts and additional circuits to perform a logic operation by adopting the majority rule and employing non-local spin signals. Also, this is in contrast to refs^20,21^ that require ancillary charge-based elements, magnetoelectric materials, in-plane and perpendicular anisotropy ferromagnetic materials, and various interfaces to perform a logic operation by relying on magnetoelectric switching mechanism and by adopting the majority rule. Furthermore, the proposals in refs.^20,21^ utilize the charge degree of freedom in performing a logic operation, thus, similar to the proposal in ref.^16^, they require frequent spin to charge conversion which compromises possible advantages of spin-based computing. Previous proposals for reconfigurable spin-based computing all require a reconfigurable magnetic setup embedded in each individual logic gate to configure the gate at run time. This requires switching of one or more magnetic contacts in a gate, and the use of extra hardware elements in the gates counteracts the advantage of reconfigurability. The proposed spin-orbitronics gate in this paper can be electrically reconfigured at run time simply by choosing the amplitude of the clock pulse.

## Structure and Operation of the Spin-Orbit Perpendicular-Anisotropy Gate

**Figure 1:**
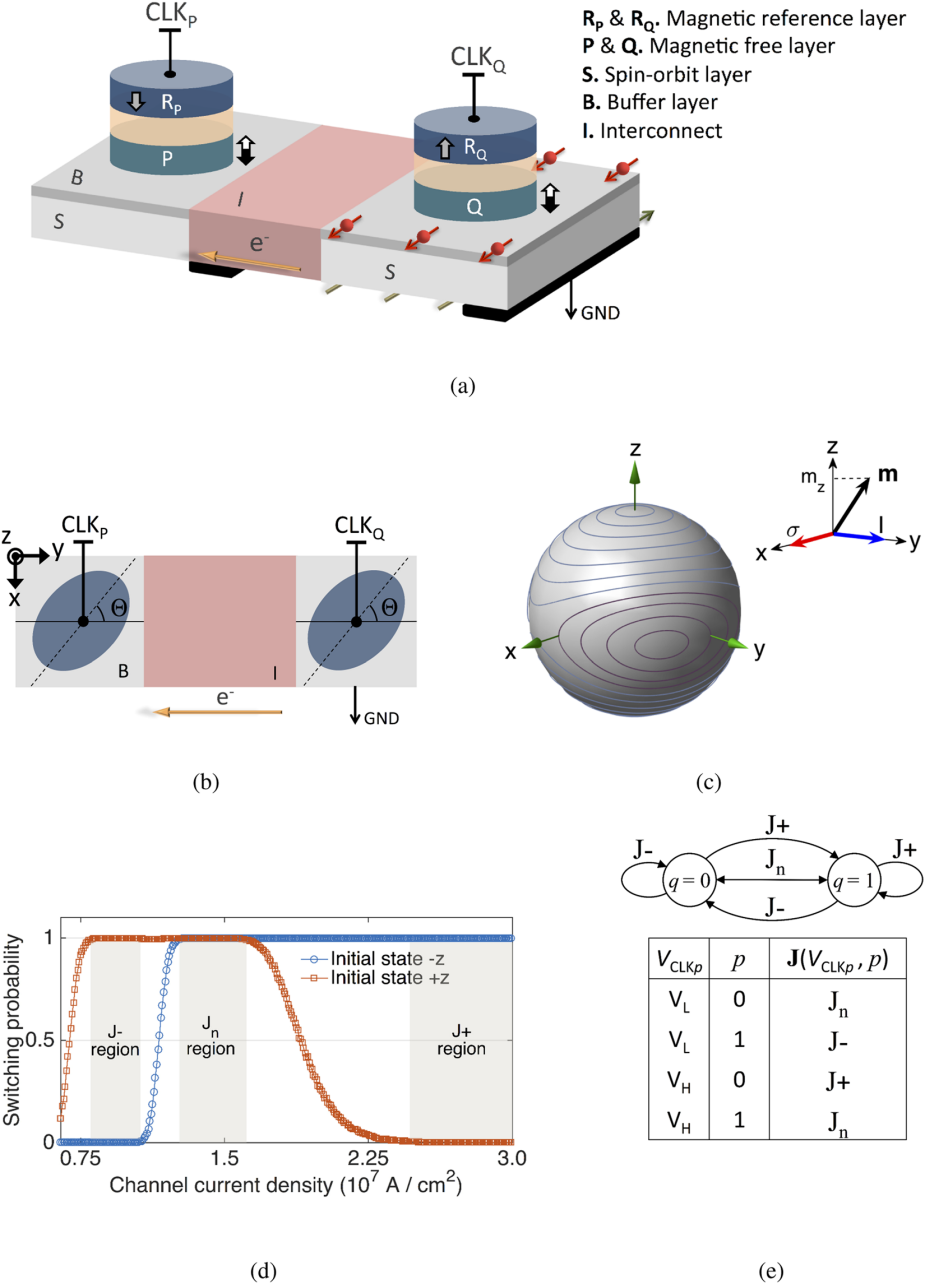
Spin-orbit perpendicular-anisotropy (SOPE) logic gate. (**a**) Logic operands are stored over the bistable magnetization state of nanomagnets *P* and *Q* which communicate through a channel. The channel comprises two segments with strong spin-orbit interaction (S) which are connected via an interconnect (I). The interconnect material can comprise a low resistivity metal such as copper, and the spin-orbit segments can be made of a heavy metal^4–10^ or a topological insulator^11–13^. The gate operates based on the ‘bounded switching’ of *Q* through the spin-orbit torques produced by a current pulse injected into the channel by applying a voltage pulse to CLK_*P*_. Information stored in *P* is used to control the current density through the magnetoresistance effect for a given amplitude of the voltage pulse applied to CLK_*P*_. (**b**) Magnets *P* and *Q* are elliptical, with the length of the ellipse enclosing an angle of Θ with the current flow (*y* axis). (**c**) The magnetic energy landscape of *P* and *Q* is asymmetric with respect to the spin accumulation direction *σ* (*x* axis), leading to the bounded switching. (**d**) Bounded switching probability diagrams of *P* or *Q* for Θ = 60° at room temperature as a function of the channel current density produced by a clock pulse with a duration of 75 ps applied to the CLK_*P*_ (see also Fig. 2). We use experimentally verified parameters^13^ of Bi_*x*_Se_(1 − *x*)_(4 nm)/Ta (0.5 nm) for the channel, where the numbers in parenthesis are the layers thicknesses. Accordingly, the spin Hall angle is set to *ζ* = 18.83. Perpendicular magnetic anisotropy (PMA) field is set to 1000 Oe, damping coefficient is set to 0.2, and the saturation magnetization is set to 2000 emu/cm^3^. The width and length of *P* and *Q* are, respectively, 32 nm and 96 nm and the channel width is 85 nm. Calculations were repeated 10,000 times for each point. (**e**) The Markov chain representing the bounded switching of *Q*. Nanomagnet *Q* retains the current state or switch to the other stable state depending on the channel current density **J** which is controlled by the magnetization state of *P* and the amplitude of the clock pulse applied to CLK_*P*_, i.e., ${\bf{J}}={\bf{J}}({{\rm{V}}}_{{\mathrm{CLK}}_{{\rm{P}}}},{\rm{P}})$).

The basic spin-orbit perpendicular-anisotropy (SOPE) gate is illustrated in Fig. 1. Perpendicular-anisotropy nanomagnets, denoted by *P* and *Q*, retain logic operands *p* and *q* over the bistable magnetization states. Magnetization orientation along +*z* (−*z*), illustrated in Fig. 1(a) by a white (black) arrow, represents binary 1 (0). As illustrated in Fig. 1(b), nanomagnets *P* and *Q* have an elliptical cross section, where the long axis of the ellipse encloses an angle of Θ with the current flow (*y* axis). Hence, the magnetic energy landscape of the nanomagnets, illustrated in Fig. 1(c), is asymmetric with respect to the spin accumulation direction *σ* (*x* axis).

**Figure 2:**
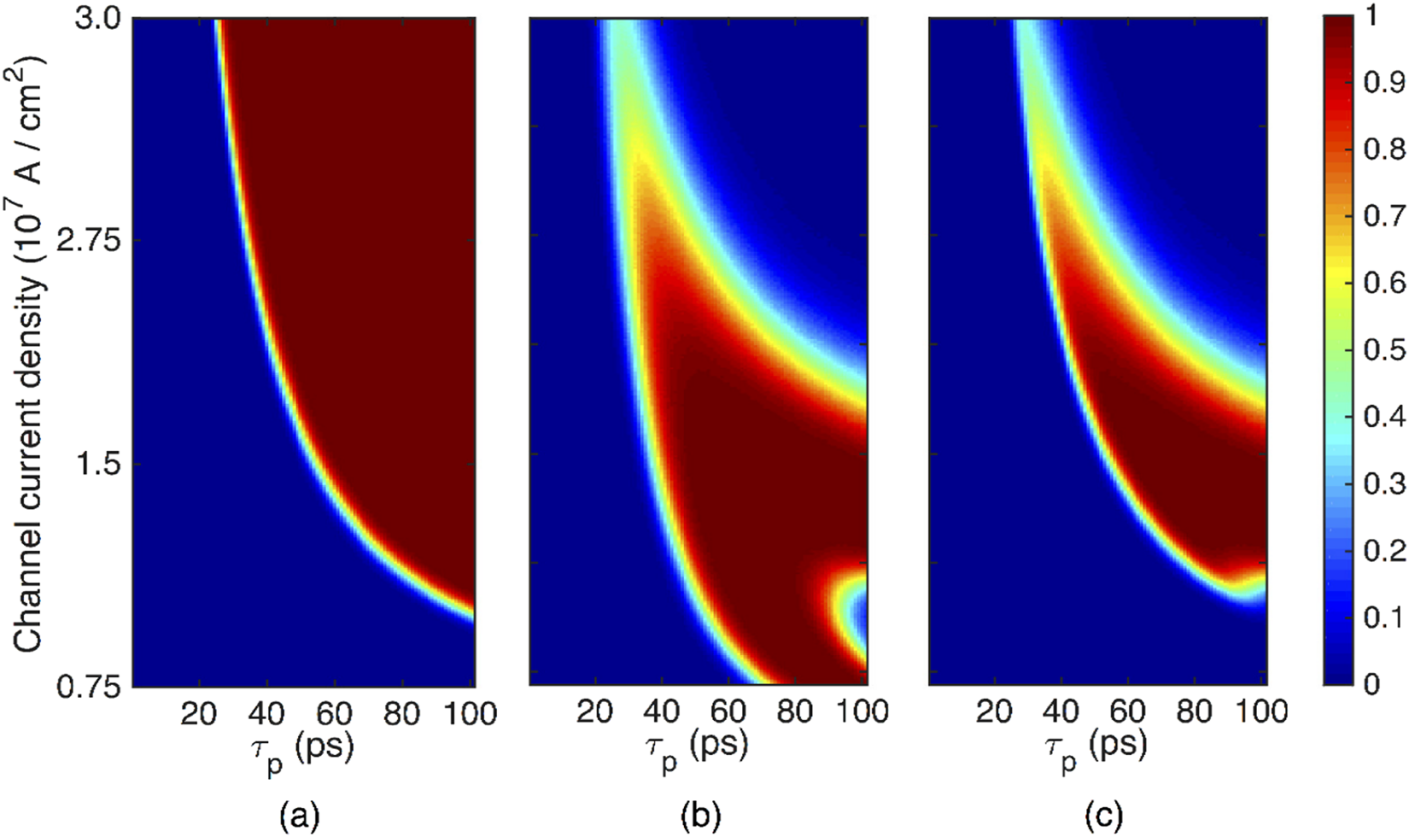
Bounded switching probability diagrams at room temperature, corresponding to the nanomagnet *P* or *Q* for Θ = 60°, as a function of the channel current density and duration (*τ*_*p*_). Device parameters are as explained in Fig. 1. (**a**) Switching probability digram corresponding to the initial magnetization state −*z*. (**b**) Switching probability digram corresponding to the initial magnetization state +*z*. (**c**) Probability digram for switching events independent of the initial magnetization state (multiplication of diagrams illustrated in Figs. 2 (a and b). The calculations were repeated 10,000 times for each pixel.

The concept underlying the operation of the proposed gate is an intrinsic property that we refer to as the ‘bounded switching’. Due to the asymmetry of the magnetic energy landscape with respect to the spin accumulation direction, as illustrated in Fig. 1(c), a channel current with a duration *τ*_*p*_ and a density J_*n*_ reverses the magnetization independent of the initial state (+*z* or −*z*), while a channel current with the same duration and a larger density J+ (smaller density J−) reverses the magnetization only if the initial state is −*z* (+*z*) [see Figs. 1 and 2]. Hence, as illustrated in Fig. 1(d), the switching probability diagram in response to current-induced spin-orbit torques comprises three primary regions denoted as J−, J_n_, and J+ region. Accordingly, as the Markov chain in Fig. 1(e) illustrates, nanomagnet *Q* may retain the initial state or switch the other state, depending on the density of the channel current produced by applying a clock pulse to CLK_*P*_. The Markov chain comprises two states representing the stable states of *Q*, i.e., *q* = 0 and *q* = 1. The *a* $\mathop{\longrightarrow }\limits^{b}$ *c* over the Markov chain is read as ‘the state of *Q* changes from *a* to *c* if the channel current density lies within the region *b*’. For a given amplitude of the clock pulse (${{\rm{V}}}_{{\mathrm{CLK}}_{{\rm{P}}}}$), operand *p* controls the channel current density (**J**) via the magnetoresistance effect.

To assess the bounded switching operation at room temperature, we performed simulations using an experimentally benchmarked model for perpendicular-anisotropy spin-orbit heterostructures^22,23^ (see Supplementary Section 1). As illustrated in Fig. 2, bounded switching is deterministically achievable in the presence of thermal noise and Joule heating for a wide range of the density and duration of the channel current. This eliminates the need for complex circuits to precisely control the duration and amplitude of the clock pulse. Hence, as in proposals for all-spin logic devices^18^, a clock pulse may be delivered to a SOPE gate through a conventional clock distribution network providing zero (ground) and nonzero voltages up to a few hundreds of millivolts and zero (high impedance) and nonzero currents up to a few hundreds of microamperes.

The bounded switching mechanism can be explained in terms of spin current interaction with the magnetic energy landscape. By modulating the magnetic energy landscape with respect to the spin accumulation direction, as illustrated in Fig. 1(c), the symmetry of the energy barrier between the two stable magnetization states is broken. Hence, depending on the initial magnetization state, different energy levels are required to switch the magnetization. These levels overlap, thereby creating an energy zone that switches the magnetization independent of the initial state. To our knowledge, bounded switching has not been observed in other spin-based or charge-based mechanisms of magnetization manipulation.

The density of the channel current produced by applying a clock pulse to CLK_*P*_ is determined by the amplitude of the clock pulse and the logic operand *p* stored in nanomagnet *P*, i.e., ${\bf{J}}={\bf{J}}({{\rm{V}}}_{{\mathrm{CLK}}_{{\rm{P}}}},{\rm{P}})$), where **J** denotes the channel current density and ${{\rm{V}}}_{{\mathrm{CLK}}_{{\rm{P}}}}$ denotes the amplitude of the clock pulse. For a given amplitude of the clock pulse, the logic operand stored in nanomagnet *P* controls the density of the channel current through the tunneling magnetoresistance effect (see Supplementary Section 2), i.e., **J**(${{\rm{V}}}_{{\mathrm{CLK}}_{{\rm{P}}}}$, *p* = 0) = **J**_0_^*p*^ and **J**(${{\rm{V}}}_{{\mathrm{CLK}}_{{\rm{P}}}}$, *p* = 1) = **J**_1_^*p*^. Since the magnetization orientation of the reference layer R_*p*_ is along the −*z*, an operand *p* = 0 (*p* = 1) leads to a parallel (antiparallel) configuration of the magnetic tunnel junction which comprises *P* and R_*P*_ (MTJ_*P*_). Thus, **J**_0_^*p*^ is larger than **J**_1_^*p*^. We use the standard notation ‘∈’ to represent the region in which the channel current density lies, e.g., **J**(${{\rm{V}}}_{{\mathrm{CLK}}_{{\rm{P}}}}$, *p*) ∈ J_*n*_ is read as ‘**J**(${{\rm{V}}}_{{\mathrm{CLK}}_{{\rm{P}}}}$, *p*) lies within the J_*n*_ region’.

**Figure 3:**
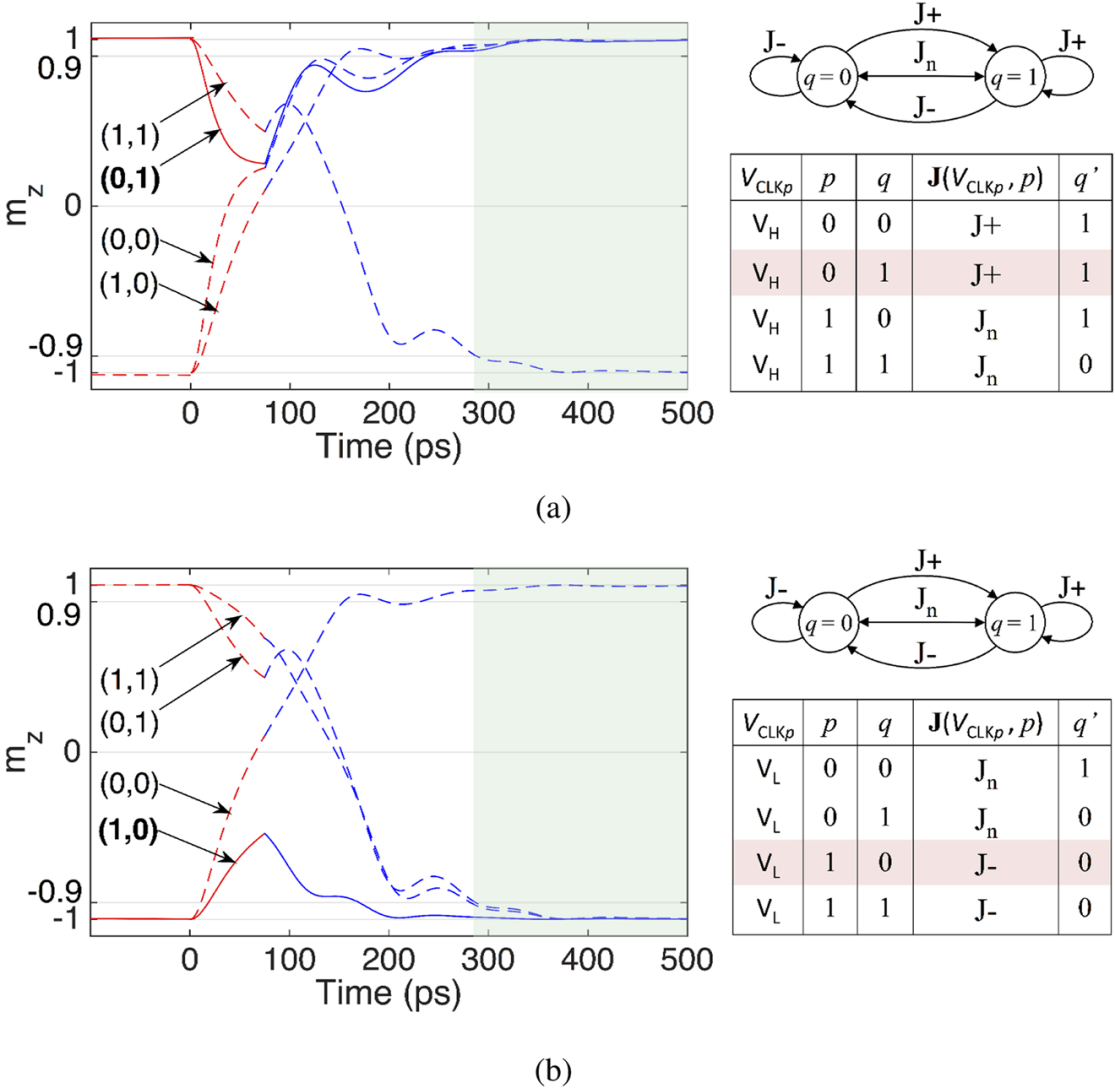
Modeled behavior of the SOPE gate for NAND and NOR operations. Time evolution of the magnetization of *Q* along the *z* axis is denoted by m_z_. (**a**) NAND operation. The amplitude of the clock pulse is set to *V*_*H*_ such that **J**(*V*_*H*_, *p* = 0) ∈ J+ and **J**(*V*_*H*_, *p* = 1) ∈ J_*n*_. Thus, the magnetization of *Q* is preserved against switching only when (*p*, *q*) = (0, 1), realizing the NAND operation in response to the clock pulse. (**b**) NOR operation. The amplitude of the clock pulse is set to *V*_L_ (<*V*_H_) such that **J**(*V*_*L*_, *p* = 0) ∈ J_*n*_ and **J**(*V*_*L*_, *p* = 1) ∈ J−. Hence, the magnetization of *Q* retains the initial state only when (*p*, *q*) = (1, 0), realizing the NOR operation in response to the clock pulse.

Once a clock pulse is applied, a current flows into the channel and evolves the magnetization state of *Q* to the result of the logic operation targeted by choosing the clock amplitude. Here we explain the execution of the NAND and NOR operations, which are known to be universal, that is, every other function can be implemented using a network of NAND or NOR gates. By setting the amplitude of the clock pulse to *V*_*H*_ such that ***J***_0_^*p*^ ∈ J+ and ***J***_1_^*p*^ ∈ J_*n*_, the magnetization orientation of *Q* is preserved against reversal only when its initial orientation is +*z* (*q* = 1) and the channel current density lies within the J+ region (*p* = 0), as illustrated in Fig. 3(a). Consequently, magnetization of *Q* evolves to *q*′ = *p*NAND*q*. Alternatively, by setting the amplitude of the clock pulse to a sufficiently smaller value *V*_*L*_ such that **J**_0_ ∈ J_n_ and **J**_1_ ∈ J−, the magnetization orientation of *Q* is preserved only when its initial orientation is −*z* (*q* = 0) and the amplitude of the channel current lies within the J− region (*p* = 1), as illustrated in Fig. 3(b). In this case, the magnetization of *Q* evolves to *q*′ = *p*NOR*q*.

## Cascadability of the Spin-Orbit Perpendicular-Anisotropy Gates

**Figure 4:**
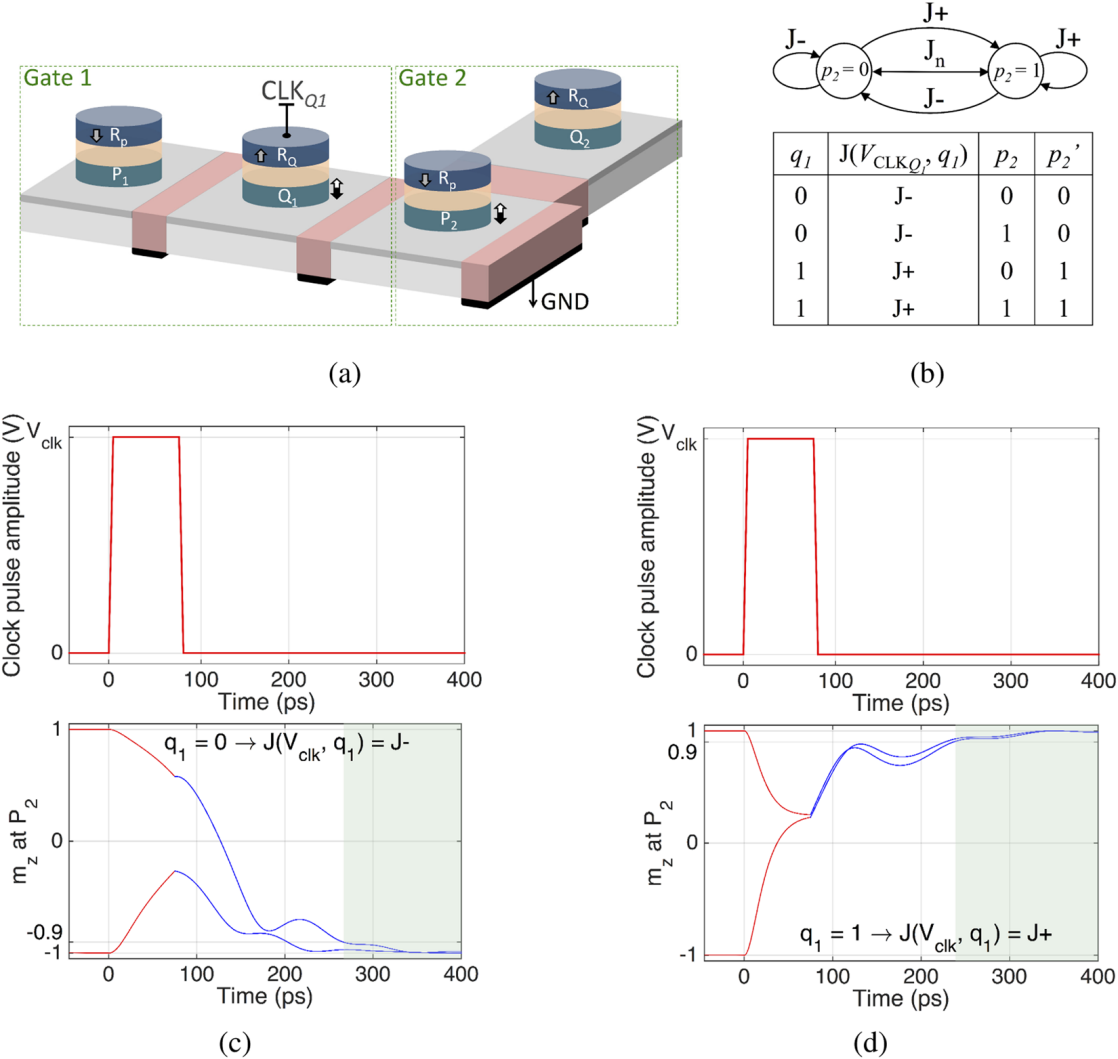
Cascading SOPE gates. Inputs and output states of a SOPE gate are represented using the stable states of a bistable magnetization, providing the opportunity to directly cascade the gates. (**a**) Two cascaded gates. (**b**) Markov chain representing state transfer from the output of Gate 1 to an input of Gate 2. By setting the amplitude of the clock pulse applied to CLK_*Q*1_ such that **J**(*V*_clk_, *q*_1_ = 0) (density of the channel current underneath *P*_2_ when *q*_1_ = 0) lies within the J− region, then **J**(*V*_clk_, *q*_1_ = 1) lies within the J+ region if TMR_*Q*1_ satisfies the constraint in equation (3). Hence, by applying the clock pulse to CLK_*Q*1_, the output state of Gate 1 (*q*_1_) is transferred to the input of Gate 2 (*P*_2_). Magnetization switching trajectories corresponding to *P*_2_, illustrating (**c**) transfer of *q*_1_ = 0 to *P*_2_ and (**d**) transfer of *q*_1_ = 1 to *P*_2_. Switching probability diagram of the gates is illustrated in Fig. 1(d) for *τ*_*p*_ = 75 ps and in Fig. 2 for a wide range of *τ*_*p*_.

The inputs and output states in a SOPE gate are represented using the stable magnetization states, providing the opportunity to directly cascade the gates without the need for any interface. Figure 4 illustrates two cascaded gates denoted as the Gate 1 and Gate 2. Each gate is a two inputs, one output gate. The output state of the Gate 1 (magnetization state of *Q*_1_) can be copied to an input of Gate 2 (nanomagnet *P*_2_) via current induced spin-orbit torques produced by applying a clock pulse to the MTJ comprising *Q*_1_ and R_*Q*_ (MTJ_*Q*1_). Since the magnetization orientation of the reference layer R_*Q*_ is along the +*z*, an operand *q*_1_ = 0 (*q*_1_ = 1) leads to an antiparallel (parallel) configuration of MTJ_*Q*1_. Thus, **J**(${{\rm{V}}}_{{\mathrm{CLK}}_{{\rm{Q1}}}}$, *q*_1_ = 0) = **J**_0_^*q*^ is smaller than **J**(${{\rm{V}}}_{{\mathrm{CLK}}_{{\rm{Q1}}}}$, *q*_1_ = 1) = **J**_1_^*q*^. The density of the channel current produced by applying a clock pulse to CLK_*Q*1_ should satisfy

1 $${\bf{J}}({V}_{{{\rm{CLK}}}_{{Q}_{1}}},{q}_{1}=\mathrm{0)}={{\bf{J}}}_{0}^{q}\in {\rm{J}}-$$

2 $${\bf{J}}({V}_{{{\rm{CLK}}}_{{Q}_{1}}},{q}_{1}=\mathrm{1)}={{\bf{J}}}_{1}^{q}\in {\rm{J}}+,$$

so that, by applying the clock pulse, the state of *P*_2_ is switched to ${p}_{2}^{\prime} =1({p}_{2}^{\prime} =0)$ only if the state of *Q*_1_ is *q*_1_ = 1 (*q*_1_ = 0), thereby performing the copy operation as illustrated in Fig. 4(b). By setting ${{\rm{V}}}_{{\mathrm{CLK}}_{{\rm{Q1}}}}$ to *V*_clk_ such that **J**_0_^*q*^ ∈ J −, the ***J***_1_^*q*^ ∈ J+ is ensured if TMR_*Q*1_ satisfy

3 $${{\rm{TMR}}}_{{Q}_{1}}\ge \frac{{R}_{eff}+{R}_{1}}{{R}_{1}}\frac{{\bf{J}}({V}_{{\rm{clk}}},{q}_{1}=\mathrm{1)}-{\bf{J}}({V}_{{\rm{clk}}},{q}_{1}=\mathrm{0)}}{{\bf{J}}({V}_{{\rm{clk}}},{q}_{1}=\mathrm{0)}},$$

where *R*_1_ denotes the resistance of MTJ_*Q*1_ when *q*_1_ = 1 and *R*_*eff*_  denotes the effective channel resistance (see Supplementary Section 3).

The resistance and TMR of an MTJ can be tuned over wide ranges. Experiments have demonstrated TMR values larger than six (600%)^24^. By designing the resistance of MTJ_*Q*1_ with respect to *R*_*eff*_, a conventional value of TMR_*Q*1_ satisfies equation (3). For the gates with switching probability diagram illustrated in Fig. 1(d), by setting ${{\rm{V}}}_{{\mathrm{CLK}}_{{\rm{Q1}}}}$such that **J**_0_^*q*^ = 10^7^ *A*/cm^2^ (∈ J−) and by designing *R*_1_ = *R*_*eff*_, a TMR = 3 (300%) leads to **J**_1_^*q*^ = 2.5×10^7^ *A*/cm^2^ that lies within the J+ region. Figures 4(c) and (d) illustrate the corresponding magnetization trajectory of *P*_2_ along the *z* axis in response to application of the clock pulse to CLK_*Q*1_.

The cascadability of the SOPE gates is significantly robust against process variations. According to equations (1) and (2), **J**_0_^*q*^ and **J**_1_^*q*^ may variate over the entire range of J− and J+ region, respectively. Hence, ${{\rm{V}}}_{{\mathrm{CLK}}_{{\rm{Q1}}}}$ and TMR_*Q*1_ may change over a wide range while satisfying constraints (1), (2) and (3). Hence, as in proposals for all-spin logic devices^18^, a clock pulse may be delivered through a conventional clock distribution network providing zero (ground) and nonzero voltages up to a few hundreds of millivolts and zero (high impedance) and nonzero currents up to a few hundreds of microamperes.

## Dynamics of Time Evolution and Switching Speed

The switching time of nanomagnets in a SOPE gate is governed by the dynamics of the magnetization evolution in response to current induced spin-orbit torques. By applying a clock pulse for performing a logic operation or transferring the gate output state to the input of another gate, current induced spin-orbit torques move the magnetization from the stable state toward the *x*–*y* plane. Once the pulse is turned off, the magnetization may either turn back to the initial stable state or switch to the other stable state, depending on the duration and amplitude of the clock pulse, as illustrated in Fig. 2. In both cases, the magnetization is stabilized through the interaction with the magnetic energy landscape of the nanomagnet. The duration of the stabilization process significantly depends on the strength of the demagnetization and PMA fields governing the magnetic energy landscape. By increasing the PMA field or decreasing the lateral dimensions of the nanomagnets, which in turn strengthen the in-plane demagnetization fields, the magnetization significantly faster evolves, resulting in a faster switching time.

**Figure 5:**
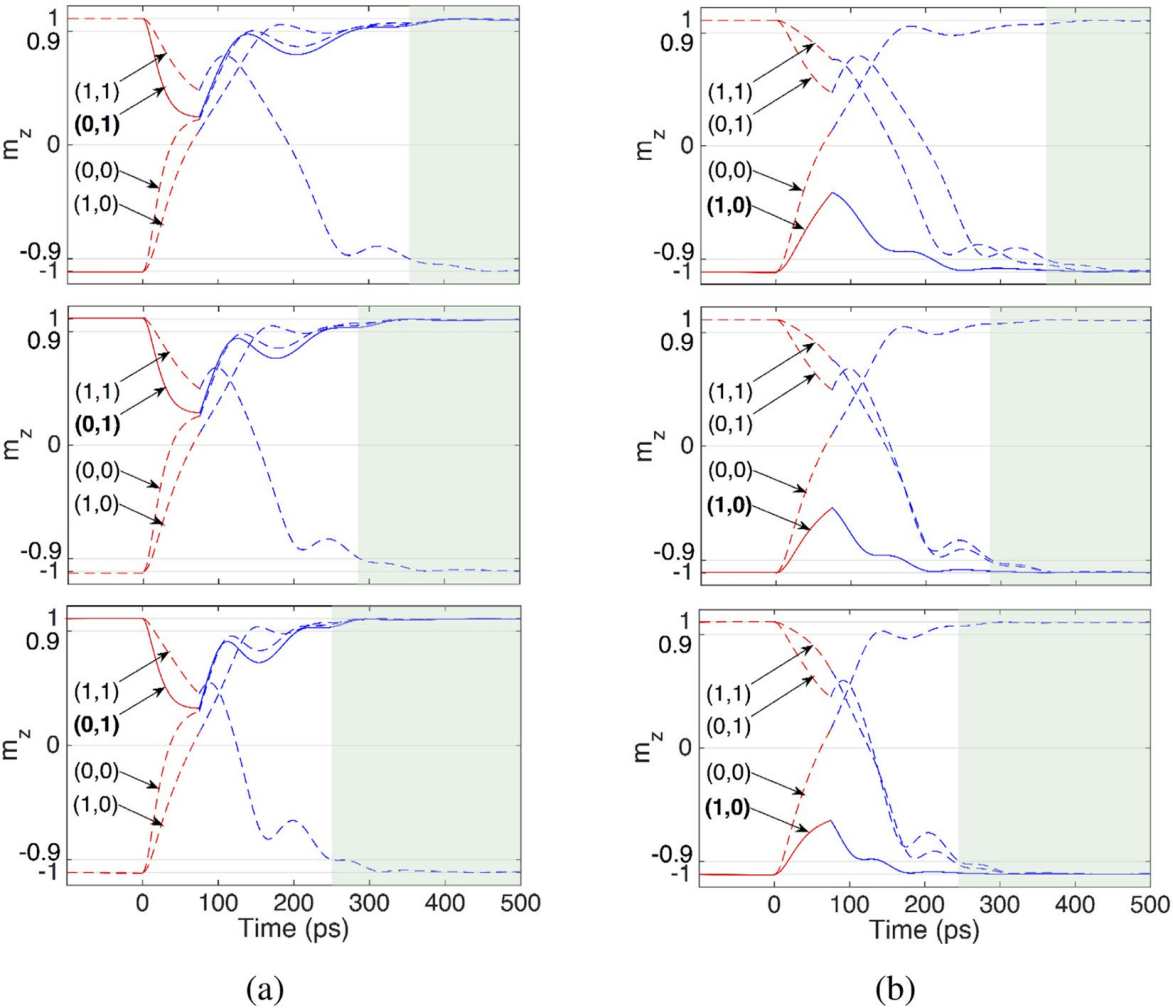
Modeled behavior of SOPE gates with different sizes and PMA fields for the NAND and NOR operations. Switching time is reduced from 365 ps to below 250 ps by increasing the PMA field from 800 Oe to 1200 Oe and reducing the size of the nanomagnets from 40 nm × 120 nm to 24 nm × 72 nm. (**a**) Magnetization switching trajectories of NAND operation performed by the gate composed of nanomagnets with, from top to bottom, size (PMA field) 40 nm × 120 nm, 32 nm × 96 nm, and 24 nm × 72 nm (800 Oe, 1000 Oe, and 1200 Oe), respectively. (**b**) Magnetization switching trajectories of NOR operation performed by the gate composed of nanomagnets with, from top to bottom, size (PMA field) 40 nm × 120 nm, 32 nm × 96 nm, and 24 nm × 72 nm (800 Oe, 1000 Oe, and 1200 Oe), respectively.

Switching dynamics corresponding to the NAND and NOR operations are illustrated in Fig. 5 for three gates composed of nanomagnets with different sizes and PMA fields. Switching trajectories, from top to bottom, correspond to the gates composed of nanomagnets with size (PMA field) 40 nm × 120 nm, 32 nm × 96 nm, and 24 nm × 72 nm (800 Oe, 1000 Oe, and 1200 Oe), respectively. Switching time is defined as the duration over which the magnetization takes 90% of the path toward an stable state. The switching time largely reduces from 365 ps to below 250 ps, which allows operations at a clock frequency larger than 4 GHz.

The other primary basis underlying the performance of a computing system are the computing architecture and resource utilization under the power dissipation constraint. The performance of a computing system based on the proposed concept in this paper is discussed in more detail within the Discussion section.

## Energy Dissipation

Energy dissipated by a SOPE gate depends on the materials and interfaces. For every electron charge injected into the channel, many units of angular momentum may flow into the ferromagnetic layer, leading to an energy efficient operation. Energy dissipation by existing spin-orbit heterostructures is more than the theoretical lower bound, and more work is required to experimentally achieve devices with energy dissipation closer to the theoretical limit. The spin torque ratio (*ζ*), that is, the strength of the damping-like spin-orbit torque per unit density of the charge current in the channel, greatly affects the energy dissipation, because this quantity fundamentally determines the current density required to perform a logic operation. The resistivity of the spin-orbit channel (*ρ*_*s*_), made of a material with strong spin-orbit interaction such as a heavy metal^4–10^ or a topological insulator^11–13^, is the other primary factor affecting the energy dissipation. It has been experimentally demonstrated^9,13^ that inserting a buffer layer between the spin-orbit layer and the ferromagnet significantly enhances *ζ* and reduces *ρ*_*s*_. The buffer layer, denoted by *B* in Fig. 1(a), can be a thin layer of pure heavy metal, graphene, or bilayer graphene. With existing experimental parameters, the energy dissipation by a SOPE gate for performing a universal logic operation ranges from a few aJ to a few fJ (see Supplementary Section 4).

## Discussion

State-of-the-art charge-based processing cores^25,26^ require frequent communication with a memory system to perform computing. This leads to the Von Neumann computing architecture, where a computing system is composed of separated processing and memory units. The access to the memory unit may take from a few nanoseconds to a few microseconds, thereby largely degrading the performance and increasing the power dissipation. Hence, a primary approach to enhance the performance and power dissipation of a computing system is to integrate more memory near the processor^25,26^. However, whereas emerging data processing and learning applications need computational resources far beyond state-of-the-art charge-based computers^27^, the opportunity to integrate more on-chip memory is largely limited as the complementary-metal-oxide-semiconductor (CMOS) technology scaling approaches the fundamental limits^3^. Furthermore, in multi-gigahertz charge-based processing cores, only a fraction of on-chip resources may be efficiently utilized without permanent damage to the system by the heat generated via high power dissipation, leading to the dark silicon phenomena^28^.

The SOPE gate is a conceptual step toward an ultra-energy efficient, reconfigurable computing system operating on a beyond Von Neumann architecture. Spin degree of freedom is utilized to enable electrically reconfigurable nonvolatile computing where the same devices retaining logic operands perform the logic operation and simultaneously retain the result, thus addressing both Von Neumann bottleneck and high power dissipation in state-of-the-art computing systems.

The size of a SOPE gate composed of nanomagnets with a width 24 nm, a length 72 nm, and Θ = 60° can be 2.5× smaller than a transistor with minimum size implemented in 14 nm FinFET CMOS technology^29^ that is state-of-the-art technology for the implementation of charge-based processors. Accordingly, the size of the SOPE gate can be more than one order of magnitude smaller than the size of a NAND or NOR gate in 14 nm FinFET CMOS Technology (See Supplementary Section 5). The ultra-small footprint of the gate results in large amount of on-chip computational resources which can be utilized effectively thanks to the low energy dissipation of the gate. This in turn could achieve a significant performance gain.

## Conclusions

We have shown that spin-orbit materials provide a natural basis for spin-based execution of logic operations. Accordingly, we proposed a spin-orbit logic gate that is electrically reconfigurable and performs a universal logic operation utilizing the minimum possible number of devices. The proposed gate is scalable to ultralow energy dissipation levels. The proposed logic scheme provides a promising approach for beyond Von Neumann spin-based computing, where the elements retaining data serve to simultaneously perform logic operations and store the result. Also, the proposed logic gate may prove beneficial in data intensive applications such as deep learning and bioinformatics, where data exchange between the storage and processing units is the primary source of energy dissipation.

## Electronic supplementary material

Supplementary Information
