# Supplementary material for: An electrically reconfigurable logic gate intrinsically enabled by spin-orbit materials
Source: Sci Rep. 2017 Nov 10;7:15358. doi: 10.1038/s41598-017-14783-1 (PMC5681507; doi:10.1038/s41598-017-14783-1)
Supplement: Supplementary file 1 — Supplementary Information [file 41598_2017_14783_MOESM1_ESM.pdf]

An electrically reconfigurable logic gate intrinsically enabled by spin-orbit materials

Supplementary Information

Mohammad Kazemi

Email: mkazemi@ece.rochester.edu

Electrical and Computer Engineering Department
University of Rochester

October 5, 2017

S1. Room temperature modeling of the Spin-Orbit Perpendicular-Anisotropy (SOPE) gate

The magnetic dynamics under the effect of the current induced spin-orbit torques at room temperature is governed by the Landau-Lifshitz-Gilbert-Slonczewski (LLGS)[1] equation,

$$\frac{d\mathbf{m}}{dt} = -\gamma\mathbf{m} \times \mathbf{H}_{eff} + \alpha\mathbf{m} \times \frac{d\mathbf{m}}{dt} + \gamma\mathbf{T}_{\mathrm{SOT}}, \tag{1}$$

where $\mathbf{m}$ is a unit vector along the magnetization, α is the Gilbert damping factor causing relaxation of the magnetization to its equilibrium state, and $\gamma = g\mu_B/\hbar$ is the gyromagnetic ratio, where μ_B is the Bohr magneton and g denotes the g-factor. At any instant of time, $\mathbf{m}$ makes an angle of ϑ with $\hat{z}$, while the plane of $\mathbf{m}$ and $\hat{z}$ makes an angle φ with $\hat{x}$. The spin-orbit torque comprises a damping-like torque and a field-like torque,

$$\mathbf{T}_{\mathrm{SOT}} = \mathbf{T}_{\mathrm{DL}} + \mathbf{T}_{\mathrm{FL}} = \frac{\hbar}{2e}\frac{J(t)}{M_s t_F}(\zeta\,\mathbf{m} \times (\boldsymbol{x} \times \mathbf{m}) + \zeta_{\perp}\mathbf{m} \times \boldsymbol{x}), \tag{2}$$

where ζ and $\zeta_{\perp}$ denote the efficiency of the current in producing the damping-like and the field-like components, respectively. Since the field-like torque may be negligible in comparison with the damping-like torque[2,3,4], the auxiliary effect of the field-like spin-orbit torque is skipped here. M_s denotes the saturation magnetization and t_F denotes the thickness of the nanomagnet placed on the spin-orbit channel. $\mathbf{H}_{eff}$ represents the effective field experienced by the magnetization and is expressed as

$$\mathbf{H}_{eff} = \mathbf{H}_k + \mathbf{H}_{dp} + \mathbf{H}_d + \mathbf{H}_L. \tag{3}$$

Here $\mathbf{H}_k$ denotes the perpendicular magnetic anisotropy field,

$$\mathbf{H}_k = 2\frac{K_u}{M_s}(\mathbf{m}.\hat{z})\hat{z}, \tag{4}$$

where K_u is the magnetic anisotropy and M_s is the saturation magnetization. $\mathbf{H}_{dp}$ is the dipole field exerted by the reference layer. $\mathbf{H}_d$ denotes the demagnetization field,

$$\mathbf{H_d}(\mathbf{m}) = -4\pi M_s(N_i \sin(\vartheta)\sin(\varphi - \Theta)\hat{\imath} + N_j \sin(\vartheta)\cos(\varphi - \Theta)\hat{\jmath} + N_z \cos(\vartheta)\hat{z}), \tag{5}$$

where N_i, N_j, and N_z are demagnetizing factors and $N_i + N_j + N_z = 1$. Here, $\hat{\imath}$ and $\hat{\jmath}$ represent the unit vectors along the length and width of the nanomagnet, respectively,

$$\hat{\imath} = -\sin(\Theta)\hat{x} + \cos(\Theta)\hat{y} \tag{6a}$$

$$\hat{\jmath} = \cos(\Theta)\hat{x} + \sin(\Theta)\hat{y}. \tag{6b}$$

Angle Θ denotes the tilt angle enclosed by the length of the nanomagnet and current flow as illustrated in Fig. 1(a).

A nonzero temperature introduces thermal fluctuations to the magnetization, which is modeled by the Langevin random field $\mathbf{H}_L = (H_{L,x}, H_{L,y}, H_{L,z})$. Each component of $\mathbf{H}_L$ follows a zero-mean Gaussian random process whose standard deviation is a function of temperature[5],

$$\delta = \sqrt{\frac{2\alpha k_B T}{\gamma M_s v_F \Delta t}}. \tag{7}$$

Here v_F is the volume of the nanomagnet, T denotes the temperature, and Δt is the duration of the constant effective thermal fluctuation field. As the current flows within the channel, the temperature increases through the Joule heating effect, and is proportional to the square of the current

$$T(I) = T_0 + kI^2, \tag{8}$$

where I is the amplitude of the current, T_0 is the temperature at zero current (room temperature), and k is the Joule heating parameter which relates the temperature to the current.

Joule heating decreases M_s and K_u as [2,4,6]

$$M_s(T) = M_{s0}(1 - \beta(T - T_0)) \tag{9a}$$

$$K_u(T) = K_{u0}(1 - \eta(T - T_0)), \tag{9b}$$

where M_{s0} and K_{u0} are, respectively, the saturation magnetization and magnetic anisotropy at temperature T_0. Coefficients β and η represent the change in, respectively, M_s and K_u as the temperature changes by $T - T_0$. Substituting (8) in (9), the saturation magnetization and magnetic anisotropy are proportional to the square of the current amplitude as, respectively,

$$M_s(T) = M_{s0}(1 - \beta k I^2) \tag{10a}$$

$$K_u(T) = K_{u0}(1 - \eta k I^2). \tag{10b}$$

S2. Channel current control for logic operations

To perform a logic operation, a clock pulse is applied to CLK_P, thereby producing a current that flows into the channel underneath Q. Depending on the bit stored in P (stable magnetization state of P), the magnetic tunnel junction comprising nanomagnets R_P and P (MTJ_P) exhibits a low or a high resistance due to the magnetoresistance effect,

$$R_1^p = R_0^p(1 + \text{TMR}_P), \tag{11}$$

where TMR_P denotes the tunneling magnetoresistance ratio of MTJ_P and R_1^p (R_0^p) is the resistance of MTJ_P when $p = 1$ ($p = 0$). Accordingly, the channel current density is

$$\mathbf{J}_{0(1)}^p \propto \frac{V_{CLK_P}}{R_{0(1)}^p + R_{eff} + R_c}. \tag{12}$$

Here V_{CLK_P} is the clock pulse amplitude, R_c is the interconnect resistance, and $R_{eff} = R_s||R_b||R_m$, where R_s, R_b, and R_m denote the resistance of the spin-orbit layer, resistance of the buffer layer, and shunt resistance of nanomagnet Q. The interconnect is made of

a low resistivity metal, such as copper, thus R_c is negligible compared to $R^p_{0(1)} + R_{eff}$. Accordingly, from (11) and (12), we have

$$\mathbf{J}^p_0 \propto \frac{V_{CLK_P}}{R^p_0 + R_{eff}} \tag{13}$$

$$\mathbf{J}^p_1 \propto \frac{V_{CLK_P}}{R^p_0 \times (1 + \text{TMR}_P) + R_{eff}}. \tag{14}$$

Therefore, to switch the channel current density between $\mathbf{J}^p_0$ and $\mathbf{J}^p_1$, TMR_P should satisfy

$$\text{TMR}_P = \frac{R^p_0 + R_{eff}}{R^p_0} \frac{\mathbf{J}^p_0 - \mathbf{J}^p_1}{\mathbf{J}^p_1}. \tag{15}$$

For the gate with the switching probability diagram illustrated in Fig. 1, $\frac{\mathbf{J}^p_0}{\mathbf{J}^p_1} = 1.75$ ensures that $\mathbf{J}^p_0$ lies within the J_n region (J+ region) when V_{CLK_P} is set such that $\mathbf{J}^p_1$ lies within the J$-$ region (J_n region). Hence, by setting $R^p_0 = R_{eff}$, TMR_P = 1.5 (150%) is sufficient to switch the channel current density between $\mathbf{J}^p_1 \in \text{J}-$ and $\mathbf{J}^p_0 \in \text{J}_n$ or between $\mathbf{J}^p_1 \in \text{J}_n$ and $\mathbf{J}^p_0 \in \text{J}+$.

S3. Channel current control for state transferring in cascaded gates

Taking the same steps as in (11) to (15), and noting that $\mathbf{J}^q_1$ is larger than $\mathbf{J}^q_0$, the TMR_{Q_1} should satisfy the following constraint so that the channel current density can be switched between $\mathbf{J}^q_0$ and $\mathbf{J}^q_1$.

$$\text{TMR}_{Q_1} \geq \frac{R_1 + R_{eff}}{R_1} \frac{\mathbf{J}^q_1 - \mathbf{J}^q_0}{\mathbf{J}^q_0}, \tag{16}$$

where R_1 is the resistance of MTJ_{Q_1} when $q_1 = 1$ and $R_{eff} = R_s||R_b||R_m$ is the channel resistance seen from Q_1. Here, R_s, R_b, and R_m denote the resistance of the spin-orbit layer, resistance of the buffering layer, and shunt resistance of nanomagnet P_2.

S4. Energy dissipation

The average energy dissipated by performing a NOR or NAND operation using the

SOPE gate is

$$E = R_{eff} I_0^2 \tau_p + (1 + \frac{\mathrm{TMR}_P}{2}) R_{eff} I_1^2 \tau_p, \tag{17}$$

where I_0 and I_1 denote the amplitude of the current produced by applying a clock pulse with a duration τ_p to CLK$_P$ when p is, respectively, 0 and 1. By setting $\mathrm{TMR}_P = 1.5$, from (13) and (14) we have

$$E = \frac{11}{7} R_{eff} I_0^2 \tau_p = \frac{11}{7} R_{eff} (k+1)^2 \frac{I_{base}^2}{\zeta^2} \tau_p. \tag{18}$$

Here, I_{base} is the *channel* current required to perform the operation when $\zeta = 1$, and $k = \frac{\rho_s/d_s + \rho_b/d_b}{\rho_m/d_m}$ is a constant that captures the effect of finite shunt resistance of nanomagnet Q, where ρ_m, ρ_s, and ρ_b (d_m, d_s, and d_b) demote the resistivity (thickness) of the ferromagnetic layer, spin-orbit layer, and buffer layer, respectively. As illustrated in Fig. S1, the energy dissipation per operation can range from a few aJ to a few fJ.

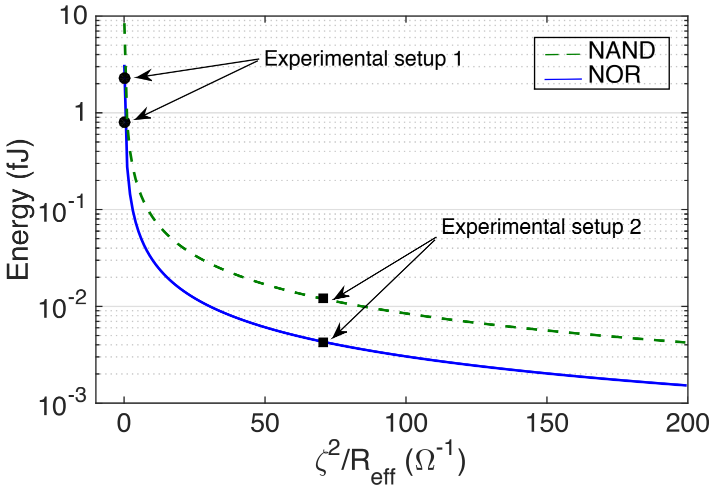

Figure S1: Energy dissipation for performing a NAND or NOR operation using the SOPE gate. The length, width, and thickness of the channel (ferromagnetic free layer) are set, respectively, to 45 nm, 65 nm, and 4 nm (72 nm, 24 nm, and 2 nm) and $\Theta = 60°$. The NAND and NOR operations are performed by a clock pulse with τ_p = 75 ps. The

marked points illustrate the energy dissipation obtained using experimentally measured parameters. For the experimental setup 1 (ref. 3), a buffer layer of Ta with a thickness of 0.5 nm is inserted between the spin-orbit channel and the ferromagnet, ζ is 18.83, and R_{eff} is 1 kΩ. For the experimental setup 2 (ref. 4), the spin Hall angle ζ and R_{eff} are 475 and 3 kΩ, respectively. For both experimental setups $k = 1$, shunting half of the injected current through the ferromagnet.

S5. The gate size

Figure S2 illustrates the layout of a standard transistor with four FINs in 14 nm FinFET CMOS technology. The size of the transistor is 212 nm $\times$ 240 nm. The size of a minimum-size transistor (a transistor with one FIN) in 14 nm FinFET CMOS technology is 212 nm $\times$ 86 nm. Accordingly, the area of a SOPE gate composed of nanomagnets with a width 24 nm, a length 72 nm, and $\Theta = 60°$, as illustrated in Fig. S3, can be 2.5$\times$ smaller than the minimum-size transistor. An standard charge-based NAND or NOR gate in CMOS technology is composed of four transistors. Hence, even by ignoring the size overhead imposed by the minimum metal pitch and spacing within a charge-based NAND or NOR gate, the area of a SOPE gate can be more than 10$\times$ smaller than a charge-based NAND or NOR gate implemented in 14 nm FinFET CMOS technology.

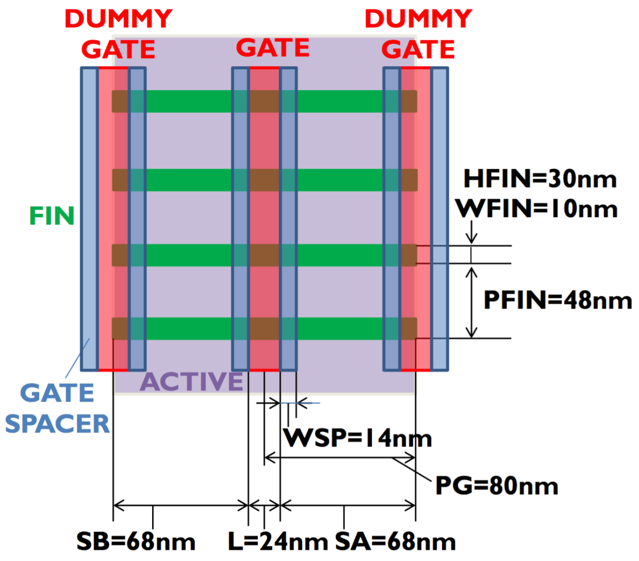

Figure S2: Layout of a standard transistor with four FINs in 14 nm FinFET CMOS technology. Nominal dimension values are indicated in the view.

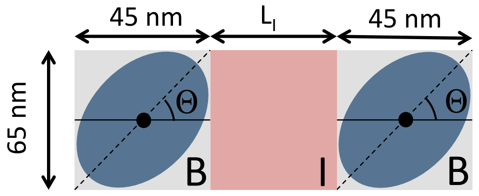

Figure S3: Top view of a SOPE gate. The length and width of the nanomagnets are set to, respectively, 72 nm and 24 nm, and $\Theta = 60°$. By setting L_I = 25 nm, the area of the SOPE gate is 2.5× smaller the minimum-size transistor in 14 nm FinFET CMOS technology.

References

1. Slonczewski, J. C. Current-driven excitation of magnetic multilayers. *Journal of Magnetism and Magnetic Materials* **159**, L1-L7 (1996).

2. Liu, L. Q., Lee, O. J., Gudmundsen, T. J., Ralph, D. C. & Buhrman, R. A. Current-induced switching of perpendicularly magnetized magnetic layers using spin torque from the spin Hall effect. *Physical Review Letters* **109**, 096602 (2012).

3. Mahendra, D. C. *et al.* "Room-temperature perpendicular magnetization switching through giant spin-orbit torque from sputtered BixSe(1-x) topological insulator material." arXiv preprint arXiv:1703.03822 (2017).

4. Fan, Y. *et al.* Magnetization switching through giant spin-orbit torque in a magnetically doped topological insulator heterostructure. *Nature Materials* **511**, 699-704 (2014).

5. Brown, W. F. Jr. Thermal fluctuations of a single-domain particle. *Journal of Applied Physics* **130**, 1677-1686 (1963).

6. Kim, J. *et al.* Layer thickness dependence of the current-induced effective field vector in Ta|CoFeB|MgO. *Nature Materials* **12**, 240-245 (2013).
